# Proteomic Identification of a Gastric Tumor ECM Signature Associated With Cancer Progression

**DOI:** 10.3389/fmolb.2022.818552

**Published:** 2022-03-01

**Authors:** Ana M. Moreira, Rui M. Ferreira, Patrícia Carneiro, Joana Figueiredo, Hugo Osório, José Barbosa, John Preto, Perpétua Pinto-do-Ó, Fátima Carneiro, Raquel Seruca

**Affiliations:** ^1^ i3S—Instituto de Investigação e Inovação em Saúde, Universidade do Porto, Porto, Portugal; ^2^ Institute of Molecular Pathology and Immunology of the University of Porto (IPATIMUP), Porto, Portugal; ^3^ Doctoral Program on Cellular and Molecular Biotechnology Applied to Health Sciences, School of Medicine and Biomedical Sciences Abel Salazar (ICBAS), University of Porto, Porto, Portugal; ^4^ Faculty of Medicine, University of Porto, Porto, Portugal; ^5^ Department of General Surgery, Centro Hospitalar Universitário de São João, Porto, Portugal; ^6^ Institute of Biomedical Engineering (INEB), University of Porto, Porto, Portugal; ^7^ School of Medicine and Biomedical Sciences Abel Salazar (ICBAS), University of Porto, Porto, Portugal; ^8^ Department of Pathology, Centro Hospitalar Universitário de São João, Porto, Portugal

**Keywords:** extracellular matrix (ECM), gastric cancer, matrisome, biomarker, proteomics

## Abstract

The extracellular matrix (ECM) plays an undisputable role in tissue homeostasis and its deregulation leads to altered mechanical and biochemical cues that impact cancer development and progression. Herein, we undertook a novel approach to address the role of gastric ECM in tumorigenesis, which remained largely unexplored. By combining decellularization techniques with a high-throughput quantitative proteomics approach, we have performed an extensive characterization of human gastric mucosa, uncovering its composition and distribution among tumor, normal adjacent and normal distant mucosa. Our results revealed a common ECM signature composed of 142 proteins and indicated that gastric carcinogenesis encompasses ECM remodeling through alterations in the abundance of 24 components, mainly basement membrane proteins. Indeed, we could only identify one *de novo* tumor-specific protein, the collagen alpha-1(X) chain (COL10A1). Functional analysis of the data demonstrated that gastric ECM remodeling favors tumor progression by activating ECM receptors and cellular processes involved in angiogenesis and cell-extrinsic metabolic regulation. By analyzing mRNA expression in an independent GC cohort available at the TGCA, we validated the expression profile of 12 differentially expressed ECM proteins. Importantly, the expression of COL1A2, LOX and LTBP2 significantly correlated with high tumor stage, with LOX and LTBP2 further impacting patient overall survival. These findings contribute for a better understanding of GC biology and highlight the role of core ECM components in gastric carcinogenesis and their clinical relevance as biomarkers of disease prognosis.

## Introduction

Gastric cancer (GC) remains a major clinical burden as one of the most reported malignancies globally, despite a steady decrease in incidence in most developed countries (Arnold et al., 2020). Strikingly, GC ranks fourth on the list of cancer-related deaths (Sung et al., 2021), reflective of its indolent nature and scarce therapeutic strategies. Indeed, surgical resection is still the cornerstone of GC treatment, however with limited effectiveness since the majority of patients are diagnosed at advanced stages of disease (Takahashi et al., 2013). Improved detection of GC, particularly at an early stage, would no doubt be crucial to ameliorate both treatment strategies and prediction of patient prognosis. In this sense, the identification of GC biomarkers has been the subject of intense investigation.

The extracellular matrix (ECM) is a key component of the gastric tumor microenvironment long overlooked as a critical source of relevant molecules for carcinogenesis (Quail and Joyce, 2013). In fact, due to its influence in cellular behavior and fate, the ECM is now regarded as a key regulator of tumorigenesis (Bonnans et al., 2014; Moreira et al., 2020), and excessive ECM deposition is considered one of the hallmarks of cancer associated with poor patient prognosis (Erler et al., 2006; Levental et al., 2009). Accordingly, the ECM provides cancer cells with sustained proliferative signals, such as growth factors and chemokines, and shields them from growth suppressors, acting as a diffusion barrier for anti-cancer conventional drugs (Henke et al., 2020). Moreover, these biochemical and mechanical changes are paramount for cancer progression since they translate into disturbed cell-cell and cell-ECM adhesion, as well as up-regulation of ECM receptors, impacting downstream signaling pathways that promote resistance to cell death, angiogenesis, cancer cell invasion and metastasis (Pickup et al., 2014; Mohan et al., 2020; Figueiredo et al., 2021).

Although known to be involved in many oncogenic processes (Pickup et al., 2014), the ECM proteome and its regulators, or “matrisome”, remained largely unexplored due to technical constraints associated to ECM protein properties, namely its high insolubility. Notably, this past decade has witnessed extensive development of tools that allow high-throughput quantitative proteomic analysis of ECM-enriched samples (Naba et al., 2012; Naba et al., 2015). This has led to the identification of matrisome protein signatures that distinguish normal tissues and primary tumors in several cancer types, including colorectal cancer (Naba et al., 2014b), triple-negative breast cancer (Naba et al., 2017) and multiple myeloma (Glavey et al., 2017). However, and to the best of our knowledge, this is the first study focusing on the gastric matrisome and on the identification of a gastric tumor ECM signature.

Herein, we undertook a comprehensive characterization of gastric ECM components following a decellularization procedure that yielded ECM enriched samples of both normal and tumor tissues. Using high-throughput proteomics, we defined the gastric matrisome and pinpointed a set of differentially expressed proteins in the tumor ECM that impact patient prognosis.

These findings contribute for the identification of molecules that play functional roles in GC development and thus can be targeted or serve as diagnostic and prognostic biomarkers.

## Materials and Methods

### Patient Samples

Human samples were obtained from the Department of Pathology from Centro Hospitalar Universitário de São João (CHUSJ, Porto, Portugal), upon patients’ informed consent. The study was approved by CHUSJ Ethics Committee for Health (Reference 32/18), in agreement with the Helsinki declaration. Gastric antrum adenocarcinoma samples, normal distant and adjacent mucosa from nine patients were collected within 1 h of surgery. The distance between adjacent normal and cancer tissue boundary was about 1 cm, while that between distant normal tissue and cancer tissue was approximately 5 cm. Samples were rinsed in ice cold PBS without calcium and magnesium, and processed into smaller fragments. When not used immediately, samples were immersed in mounting medium for cryotomy (OCT compound, Thermo Fisher Scientific, Germany), frozen in 2-methylbutane cooled with liquid nitrogen, and stored at −80°C. Patient clinicopathological data is summarized in Supplementary Table S1.

### Decellularization and ECM-Enrichment

Normal and tumor samples were cut into 4 × 4 mm fragments, weighed, placed in a 24-well plate, and decellularized as previously described (Pinto et al., 2017). Briefly, samples were incubated in a hypotonic buffer (10 mM Tris-HCl, 0.1% EDTA, pH 7.8) for 18 h, washed with PBS, and decellularized for 24 h in 0.1% SDS. Fragments were washed (10 mM Tris-HCl, pH 7.8) and incubated for 3 hours at 37°C with 50 U/ml DNase (Gibco, Thermo Fisher Scientific, Germany) in reaction buffer (10 mM Tris-HCl, 2.5 mM MgCl2, 100 nM CaCl_2_, pH 7.8). All steps were performed under constant agitation (165 rpm) in the presence of 10 mg/ml of Gentamicin (Gibco, Thermo Fisher Scientific, Germany). DNA content of native and decellularized tissues was quantified on a Qubit Fluorometer (Thermo Fisher Scientific, Germany) using the Qubit dsDNA High Sensitivity kit (Thermo Fisher Scientific, Germany), according to the manufacturer’s instructions. For nuclei staining with DAPI, one fragment from each sample was formalin-fixed and paraffin-embedded. To confirm decellularization efficiency, 3 μm-thick sections from FFPE samples were processed and stained with Hematoxylin and Eosin (H&E), as well as through Masson’s Trichrome (MT) protocol. Images were obtained on a Light microscope Olympus DP 25 (Olympus, United States).

### Sample Preparation and Liquid Chromatography-Tandem Mass Spectrometry

Normal and tumor decellularized fragments (ca. 30 mg/sample) were prepared for mass spectrometry analysis following an adapted protocol (Naba et al., 2015). Samples were homogenized on ice with a tissue ruptor in an urea solution with reducing agent dithiothreitol (DTT), alkylated with iodoacetamide, and subsequently digested with PNGaseF and trypsin/Lys-C.

Protein identification and quantitation was performed by nanoscale liquid chromatography with tandem mass spectrometry (nano LC-MS/MS) using an Ultimate 3,000 liquid chromatography system coupled to a Q-Exactive Hybrid Quadrupole-Orbitrap mass spectrometer (Thermo Fisher Scientific, Germany), as previously described (Osório et al., 2021). Data was acquired using Xcalibur 4.0 and Tune 2.09 software (Thermo Fisher Scientific, Germany).

### Data Analysis

Raw data was processed using Proteome Discoverer 2.4.0.305 software (Thermo Fisher Scientific, Germany) and submitted as query in the UniProt database for the reviewed Homo sapiens Proteome 2020_02 with 20,350 entries and the NIST human spectral library. A common protein contaminant list from MaxQuant was also considered. The MSPepSearch and Sequest HT search engines were used to identify tryptic peptides. The ion mass tolerance was 10 ppm for precursor ions and 0.02 Da for fragment ions in both software. Maximum number of missed cleavage sites allowed was set to two. Peptide confidence was set to high. The processing node Percolator was enabled with the following settings: maximum delta Cn 0.05, decoy database search target FDR 1%, and validation based on q value. Protein label free quantitation was performed with the Minora feature detector node at the processing step. Precursor ions quantification was performed considering unique plus razor peptides with the following parameters: precursor abundance based on intensity, and normalization based on total peptide amount. Concerning post-translational modifications, we have considered static modifications, such as Carbamidomethylation (cysteine), and dynamic modifications, including Oxidation (methionine) and N-terminal modifications (Acetyl, Met-loss and Met-loss + Acetyl). For protein identification, at least two unique peptides and an FDR level of confidence of “high” (*p* ≤ 0.01) were required. Single unique peptide identifications were included only when its amino acid sequence was exclusively related to the identified protein [sequences were queried using the UniProt Blast tool (Pundir et al., 2016)]. Matrisome annotations were identified using MatrisomeDB [http://matrisomedb.pepchem.org/; (Shao et al., 2019)]. Pairwise correlations of protein abundance for the same tissue across different patients were quantified using the Pearson’s correlation coefficient. The Overlap Coefficient was used to calculate the similarity between qualitative profiles obtained for each of the datasets. To define the matrisome composition of each tissue, we have only considered core ECM proteins and ECM-associated proteins that were present in at least a third of patient samples.

### Identification of Differentially Expressed Proteins

For the identification of differentially expressed matrisome proteins among tumor and normal paired samples, we first performed a multiple two-tailed Student’s *t* test corrected for multiple comparisons using the Benjamini-Hochberg method. Next, we have calculated fold change (FC) values for each protein (protein expression in tumor samples normalized against protein expression in normal distant or normal adjacent tissues). For further analysis, we have selected proteins with a *q* value <0.05 and a FC ≥ 1.5 (log_2_ FC ≥ 0.585).

### Functional and Pathway Enrichment Analysis

STRING v11.0 (Search Tool for the Retrieval of Interacting Genes/Proteins database) online database [https://string-db.org/; (Szklarczyk et al., 2019)] was used to identify functional systems or pathways overrepresented in our sets, when compared with the software’s default background (Whole genome, *Homo sapiens*). Three classification networks were considered, including gene ontology domains [“Biological Process”, “Molecular Function” and “Cellular Component” (Ashburner et al., 2000; Gene Ontology Consortium., 2021)], InterPro Protein Domains and Features (Mitchell et al., 2019; Blum et al., 2020), and KEGG pathways [Kyoto Encyclopedia of Genes and Genomes; (Kanehisa et al., 2016)]. Significance threshold was set to FDR adjusted *p* value < 0.01. Enrichment strength for each category, which refers to log_10_ values of the ratio between observed proteins from our network and the number of proteins expected to be annotated with the term in a random network of the same size, was calculated using the STRING software.

### Analysis of mRNA-Seq Data From the TCGA

The mRNA-Seq data of 27 tumor and matched normal pairs from The Cancer Genome Atlas (TCGA) stomach adenocarcinoma cohort (STAD) were plotted and compared using the TNMplot platform [https://tnmplot.com/analysis/; (Bartha and Győrffy, 2021)]. Comparisons were performed using the Wilcoxon test with a statistical significance cutoff set at *p* < 0.05. Candidates presenting tumor/normal expression ratios similar to those identified in our proteomic data were selected, and their association with clinicopathological features and overall survival of GC patients was further analysed.

For this purpose, RNA-seq data and clinical metadata of 383 STAD cases were retrieved from the cBioportal platform [https://www.cbioportal.org/study/summary?id=stad_tcga_pan_can_atlas_2018; (Cerami et al., 2012; Gao et al., 2013)] and from the supplemental data of TCGA STAD Pan (Cancer Genome Atlas Research Network., 2014; Liu et al., 2018), respectively.

To evaluate the association of ECM genes with tumor progression, patients were divided into four groups according to the Tumor Node Metastasis (TNM) grading system (I, *n* = 51; II, *n* = 115; III, *n* = 162; and IV, *n* = 39). Association with tumor histological type was studied for the 238 patients with available data. Using the Laurén classification system, patients were grouped into two main categories: diffuse type (*n* = 77) and intestinal type (*n* = 161) GC. Gene expression was compared using One-way ANOVA followed by post-hoc Tukey’s Honest Significant Difference test to perform multiple comparisons between groups of samples, or unpaired t test. Significant differences were considered when *p* < 0.05.

Using Kaplan-Meier survival analysis, GC patients were divided into two groups (high and low), according to median expression of ECM genes. Overall survival of the two groups was compared with the log rank test (Mantel-Cox) and a *p* < 0.05 was considered statistically significant.

### Statistical Analysis

To determine significant differences among independent groups, we have used parametric tests, such as Student’s *t* test or One-way ANOVA, followed by multiple comparison with post hoc correction (when applicable). A *p* value below 0.05 was considered statistically significant. To evaluate normality of the distribution, we applied the Kolmogorov-Smirnov test. Equality of group variances was verified using the Brown-Forsythe test. All analysis were carried out using GraphPad Prism (version 6.05) or IBM SPSS Statistics version 26.

## Results

In this study, we aimed to uncover the role of ECM in gastric carcinogenesis and its potential as a biomarker for cancer monitoring and surveillance. For that purpose, we have characterized the gastric matrisome evaluating both distant and adjacent mucosa, as well as tumor samples from gastric cancer patients.

### Tumor and Normal Gastric ECM Share Core Matrisome Components

To characterize the ECM composition of gastric mucosa and potential alterations that may be associated with carcinogenesis, we performed quantitative proteomic analysis of normal distant (ND), normal adjacent (NA) and tumor (T) decellularized ECM samples from nine GC patients (Supplementary Table S1).

**FIGURE 1:**
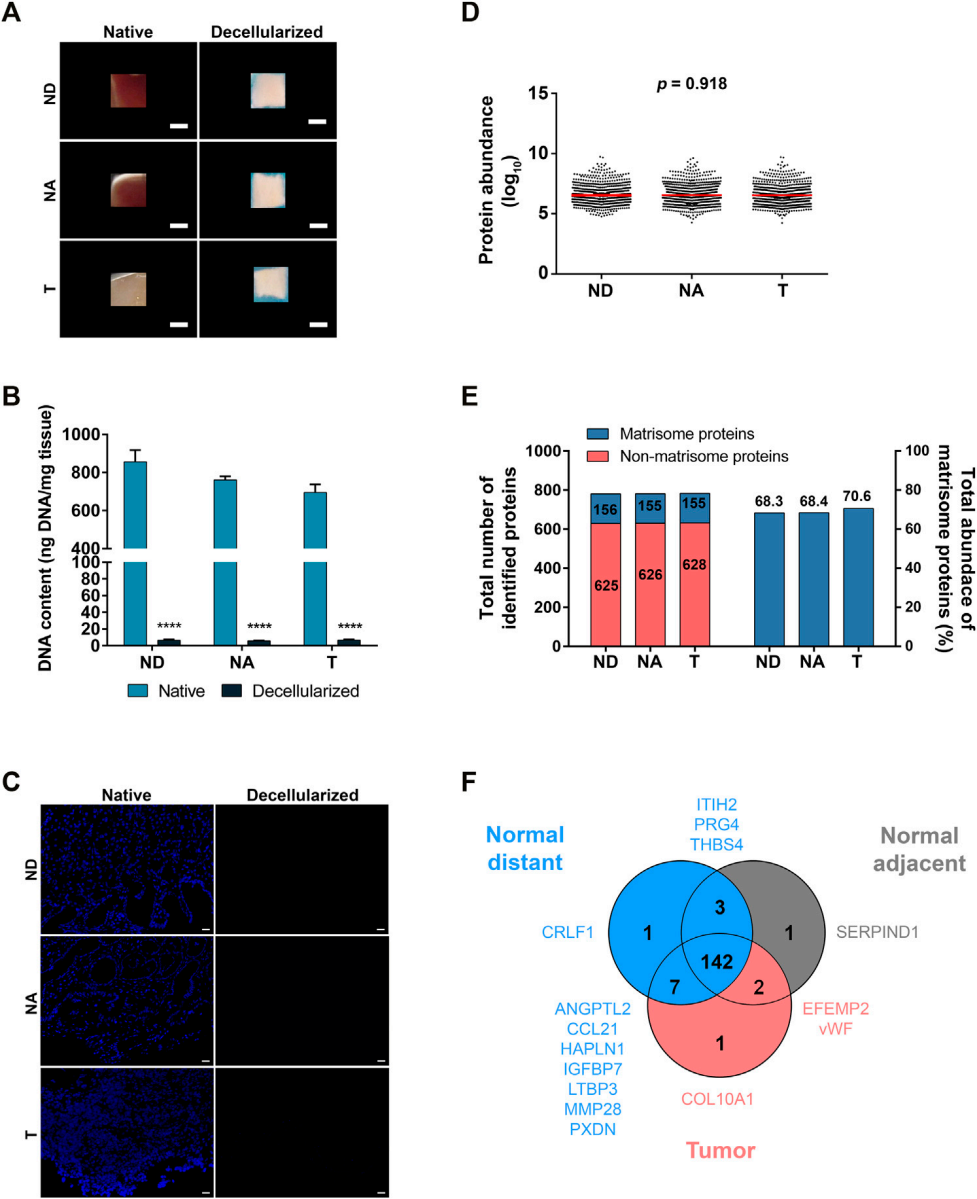
Characterization and proteomic analysis of decellularized gastric ECM. **(A)** Representative macroscopic images of normal distant (ND), normal adjacent (NA) and tumor (T) samples from human gastric mucosa before and after decellularization; scale bar = 1 mm. **(B)** DNA quantification of native and decellularized ECM samples (*n* = 3). Data are shown as mean ± SD, ****p < 0.0001. **(C)** Representative images of DAPI staining of native and decellularized samples; scale bar = 20 µm. **(D)** Log_10_ of normalized abundance distribution for all identified proteins in decellularized samples. **(E)** Total number of matrisome and non-matrisome proteins identified in decellularized ECM, with the corresponding abundance (%) of each set of matrisome proteins. **(F)** Venn diagram with the number of matrisome proteins identified in each tissue in at least 1/3 of the samples (*n* = 9). We have identified a gastric matrisome signature composed of 153 proteins (in blue) and a tumor-specific ECM signature with three proteins (in red). Proteins present in only one or two ECM sets are specified.

To obtain an ECM-enriched preparation, we undertook a three-step decellularization protocol using a hypotonic buffer, an anionic surfactant and DNAse treatment. As depicted in Figure 1A, a change in the color of gastric tissues was indicative of removal of cellular components. Decellularization efficiency was evaluated through DNA quantification and DAPI staining. Specifically, we observed nuclei loss in decellularized tissues and total DNA decreased by over 99% in normal distant (855.6 ng/mg in native vs. 5.8 ng/mg in decellularized tissue), normal adjacent (760.9 ng/mg in native vs. 6.5 ng/mg in decellularized tissue) and tumor tissues (695.5 ng/mg in native vs. 6.8 ng/mg in decellularized tissue) (Figures 1B,C). Further, we performed Hematoxylin and Eosin (H&E), as well as Masson’s Trichrome (MT) staining, confirming loss of cellular components and maintenance of ECM structure (Supplementary Figure S1).

Subsequent analysis by LC-MS/MS revealed that there was no sample preparation bias given the similar normalized abundance distribution among tissue types (Figure 1D). We have identified a total of 781 proteins both in ND and in NA mucosa, and 783 proteins in T samples (Supplementary Material S1). Bioinformatic analysis and filtering through MatrisomeDB, a comprehensive database platform for ECM-derived protein identification, indicated that only 20% of these proteins were core matrisome (ECM glycoproteins, proteoglycans and collagens) or ECM-associated proteins (secreted factors, ECM regulators and ECM-affiliated proteins). Notwithstanding, they represent 68–70% of total protein abundance (Figure 1E). Analysis of matrisome proteins in biological replicates across the three tissues revealed they are highly correlated, with average Pearson correlation coefficients for protein abundance of 0.78, 0.83 and 0.88 for ND, NA and T samples, respectively, and an overlap coefficient of 0.94, which suggests both technical reproducibility and low inter-patient variability (Supplementary Figure S2; Supplementary Material S2).

To define each tissue’s matrisome, we considered only core matrisome and ECM-associated proteins that were present in at least a third of patient samples. Accordingly, the matrisome of normal distant mucosa was found to be composed of 153 proteins and that of normal adjacent tissue of 148 proteins, whereas the tumor matrisome comprised 152 proteins. When comparing matrisome proteins of the three tissues (Figure 1F and Supplementary Figure S3), we have identified a set of 142 common and 11 more proteins that were present in the normal distant mucosa alone or in combination with one of the other tissues. The glycoproteins EGF-containing fibulin-like extracellular matrix protein 2 (EFEMP2) and von Willebrand factor (vWF) were present in tumor and normal adjacent tissue but not in the normal distant mucosa matrisome and, surprisingly, there was only one tumor-specific protein, collagen alpha-1(X) chain (COL10A1).

Significant post-translational modifications were not identified, with the exception of Met-loss + Acetyl of LGALS4, which was enriched in normal distant when compared with normal adjacent mucosa.

Overall, these results revealed a similar protein composition between normal and tumor ECM, suggesting that ECM remodeling is mostly linked to alterations in the levels of its components, rather than to qualitative changes of the components themselves.

### Tumor ECM Presents a Distinct Protein Expression Signature

**FIGURE 2:**
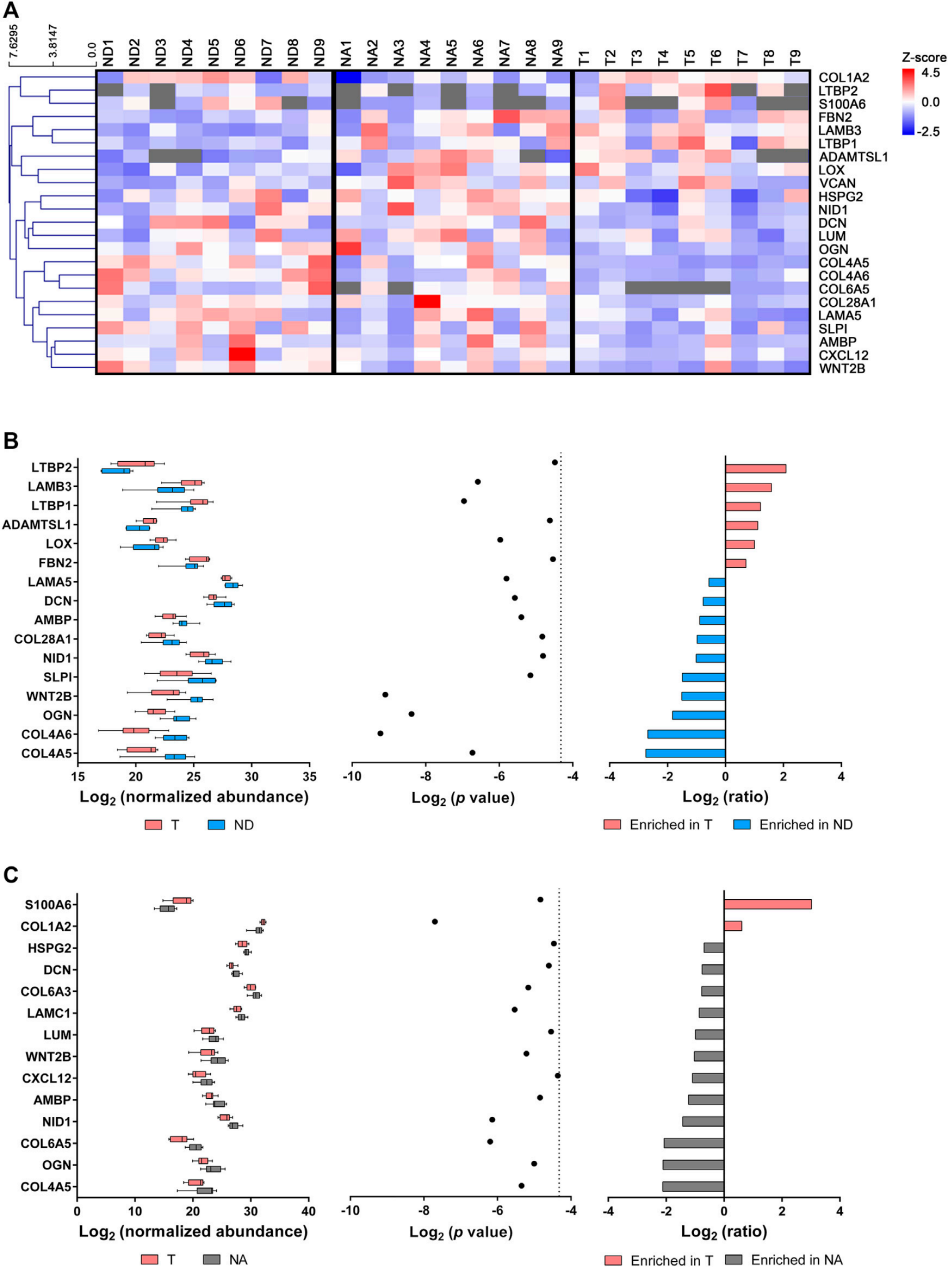
Differentially expressed proteins in gastric ECM. **(A)** Heatmap representation of hierarchical clustering (average linkage and Euclidean metric) of protein expression profiles indicated as log_2_ (normalized abundance). Grey rectangles represent expression below detection limit. Tumor (T) ECM (*n* = 9) yielded six upregulated and 10 downregulated proteins in comparison with normal distant (ND) mucosa **(B)**, while two were upregulated and 12 were downregulated when compared with normal adjacent (NA) mucosa **(C)**. Normalized abundance values were compared using a two-tailed, unpaired Student’s *t*-test corrected for multiple comparisons with the Benjamini-Hochberg method. Dotted line represents statistical significance threshold (*p* < 0.05).

Given that most ECM proteins were ubiquitously expressed in normal and tumor tissues, we next aimed to disclose molecular alterations at the protein expression level associated with GC. For that purpose, we focused on the established matrisome lists and determined differentially expressed proteins among the three tissues (Table 1 and Figure 2A).

**TABLE 1:** Differentially expressed proteins among tumor, normal distant and normal adjacent tissues.

Protein	Gene symbol	ECM class	Log_2_ Fold Change	*q* value
**Tumor vs. Normal distant**				
Latent-transforming growth factor beta-binding protein 2	LTBP2	ECM Glycoproteins	2.09	0.0029
Laminin subunit beta-3	LAMB3	ECM Glycoproteins	1.59	0.0013
Latent-transforming growth factor beta-binding protein 1	LTBP1	ECM Glycoproteins	1.21	0.0016
ADAMTS-like protein 1	ADAMTSL1	ECM Regulators	1.12	0.0020
Protein-lysine 6-oxidase	LOX	ECM Regulators	1.00	0.0026
Fibrillin-2	FBN2	ECM Glycoproteins	0.71	0.0072
Laminin subunit alpha-5	LAMA5	ECM Glycoproteins	−0.58	0.0033
Decorin	DCN	Proteoglycans	−0.77	0.0052
Protein AMBP	AMBP	ECM Regulators	−0.89	0.0131
Collagen alpha-1(XXVIII) chain	COL28A1	Collagens	−0.98	0.0042
Nidogen-1	NID1	ECM Glycoproteins	−1.01	0.0056
Antileukoproteinase	SLPI	ECM Regulators	−1.49	0.0023
Protein Wnt-2b	WNT2B	Secreted Factors	−1.51	0.0036
Osteoglycin	OGN	Proteoglycans	−1.83	0.0010
Collagen alpha-6(IV) chain	COL4A6	Collagens	−2.68	0.0003
Collagen alpha-5(IV) chain	COL4A5	Collagens	−2.75	0.0007
**Tumor vs. Normal adjacent**				
Protein S100-A6	S100A6	Secreted Factors	3.02	0.0003
Collagen alpha-2(I) chain	COL1A2	Collagens	0.61	0.0016
Basement membrane-specific heparan sulfate proteoglycan core protein	HSPG2	Proteoglycans	−0.69	0.0082
Decorin	DCN	Proteoglycans	−0.76	0.0026
Collagen alpha-3(VI) chain	COL6A3	Collagens	−0.77	0.0007
Laminin subunit gamma-1	LAMC1	ECM Glycoproteins	−0.86	0.0023
Lumican	LUM	Proteoglycans	−0.99	0.0036
Protein Wnt-2b	WNT2B	Secreted Factors	−1.03	0.0010
Stromal cell-derived factor 1	CXCL12	Secreted Factors	−1.09	0.0144
Protein AMBP	AMBP	ECM Regulators	−1.23	0.0039
Nidogen-1	NID1	ECM Glycoproteins	−1.43	0.0062
Collagen alpha-5(VI) chain	COL6A5	Collagens	−2.07	0.0020
Osteoglycin	OGN	Proteoglycans	−2.11	0.0056
Collagen alpha-5(IV) chain	COL4A5	Collagens	−2.12	0.0013
**Normal distant vs. Normal adjacent**				
Collagen alpha-6(IV) chain	COL4A6	Collagens	1.39	0.0013
Latent-transforming growth factor beta-binding protein 1	LTBP1	ECM Glycoproteins	−0.73	0.0023
Fibrinogen beta chain	FGB	ECM Glycoproteins	−0.80	0.0036
EMILIN-1	EMILIN1	ECM Glycoproteins	−0.90	0.0007
Versican core protein	VCAN	Proteoglycans	−1.01	0.0029
Laminin subunit alpha-4	LAMA4	ECM Glycoproteins	−1.19	0.0010

When comparing tumor and normal distant mucosa, we identified 16 differentially expressed proteins, six of which were upregulated and 10 were downregulated in tumors (Figure 2B). Specifically, the upregulated components in tumor tissue were: glycoproteins latent-transforming growth factor beta-binding protein 1 (LTBP1) and latent-transforming growth factor beta-binding protein 2 (LTBP2), fibrillin-2 (FBN2), laminin subunit beta-3 (LAMB3), as well as ECM regulators lysyl oxidase (LOX) and ADAMTS-like protein 1 (ADAMTSL1). Regarding downregulated tumor ECM constituents, we identified the basement membrane components collagen alpha-5(IV) chain (COL4A5), collagen alpha-6(IV) chain (COL4A6), collagen alpha-1 (XXVIII) chain (COL28A1), laminin subunit alpha-5 (LAMA5), nidogen 1 (NID1), proteoglycans osteoglycin (OGN) and decorin (DCN), ECM regulators alpha-1-microgobulin/bikunin precursor (AMBP) and secretory leucocyte protease inhibitor (SLPI), along with secreted factor protein Wnt-2b (WNT2B).

The comparison between tumor tissue and normal adjacent mucosa yielded two upregulated and twelve downregulated proteins in tumors (Figure 2C). Collagen alpha-2 (I) chain (COL1A2) and secreted factor S100 calcium binding protein A6 (S100A6) were found upregulated in GC, whereas downregulated proteins included collagen alpha-5 (VI) chain (COL6A5) and collagen alpha-3 (VI) chain (COL6A3), heparan sulfate proteoglycan 2 (HSPG2), lumican (LUM), laminin subunit gamma-1 (LAMC1) and secreted factor stromal cell-derived factor 1 (CXCL12), along with COL4A5, WNT2B, NID1, OGN, AMBP and DCN, which had been already identified when comparing tumor with normal distant mucosa.

Interestingly, when comparing normal adjacent and distant tissues, we uncovered a set of differentially expressed proteins that may represent a pro-oncogenic signature. In fact, we were able to detect five upregulated proteins in the normal mucosa adjacent to the tumor, namely laminin subunit alpha-4 (LAMA4), elastin microfibril interfacer 1 (EMILIN1), LTBP1 and fibrinogen beta chain (FGB), as well as proteoglycan versican core protein (VCAN). In contrast, COL4A6 was the only protein found downregulated in this context (Supplementary Figure S4).

Taken together, these data suggest that there is a remodeling of the ECM during GC development, encompassing changes in the abundance of matrix components, particularly of basement membrane proteins, which can impact tumor cell behavior.

### Functional Analysis Identifies Main Biological Traits Associated With Structural Remodeling of the ECM

**FIGURE 3:**
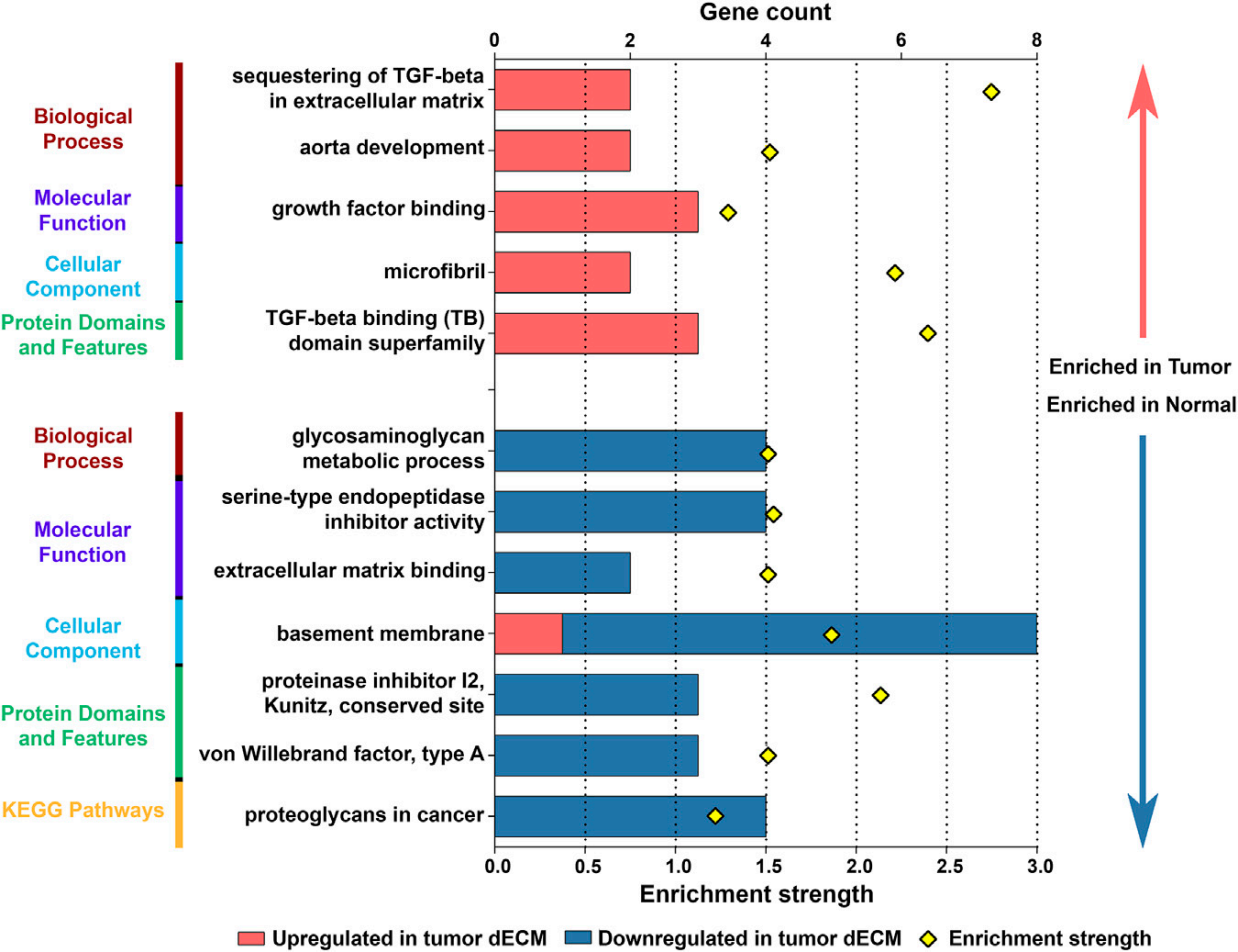
Functional and pathway enrichment analysis of differentially expressed proteins in tumor ECM. For GO term analysis, proteins were assigned to three main categories: Biological Process, Molecular Function and Cellular Component. The main Protein Domains and Features and KEGG Pathways were also identified. Graph depicts the most relevant results for each category enriched in tumor and normal ECM (additional data in Supplementary Table S2). Bars represent gene count and dots depict enrichment strength.

In order to uncover the biological significance of the set of differentially expressed ECM proteins in the tumor tissues, when compared with normal distant and adjacent mucosa, these were submitted to functional and pathway enrichment analysis (Figure 3 and Supplementary Table S2).

Through functional analysis, we found that biological processes related with sequestering of TGF-beta in the ECM (LTBP1 and FBN2; *p* = 1.10e-03) and aorta development (LOX and LTBP1; *p* = 2.99e-02), growth factor binding functions (LTBP1, LTBP2 and COL1A2; *p* = 4.40e-03), and microfibril components (LTBP1 and FBN2; *p* = 3.40e-04) were particularly enriched in upregulated proteins. Likewise, TGF-beta binding (TB) domain superfamily (LTBP1, LTBP2, FBN2; *p* = 4.76e-06) was over-represented in upregulated tumor ECM proteins. On the contrary, glycosaminoglycan metabolic processes, such as catabolism and biosynthesis (DCN, OGN, LUM and HSPG2; *p* ≤ 0.02), and molecular functions related to inhibition of enzymatic activity, such as serine-type endopeptidase inhibitor activity (AMBP, COL6A3, COL28A1, SLPI *p* = 2.2e-04), and extracellular matrix binding (DCN, NID1; *p* = 0.0151) were only correlated with downregulated proteins. Further, there was an over-representation of basement membrane components (e.g., NID1, LAMB3, HSPG2, COL4A5 and COL4A6-00; *p* = 3.64e-12), as well as proteinase inhibitors with Kunitz (AMBP, COL6A3, COL28A1; *p* = 1.56e-05) and von Willebrand Factor type A (COL6A3, COL6A5, COL28A1; *p* = 4.90e-04) domains in the downregulated class. Significantly enriched KEGG pathways, such as proteoglycans in cancer (DCN, LUM, WNT2B, HSPG2; *p* = 3.60e 04), were also mainly associated with downregulated proteins.

Collectively, the data suggest that gastric ECM remodeling favors tumor progression by activating ECM receptors and cellular processes that are involved in angiogenesis and cell-extrinsic metabolic regulation.

### ECM Alterations Associate With Gastric Cancer Progression and Overall Survival

To validate the ECM changes identified in our proteomic survey, we used RNA-seq data from normal and paired tumor gastric carcinoma samples available at the TCGA-STAD cohort.

**FIGURE 4:**
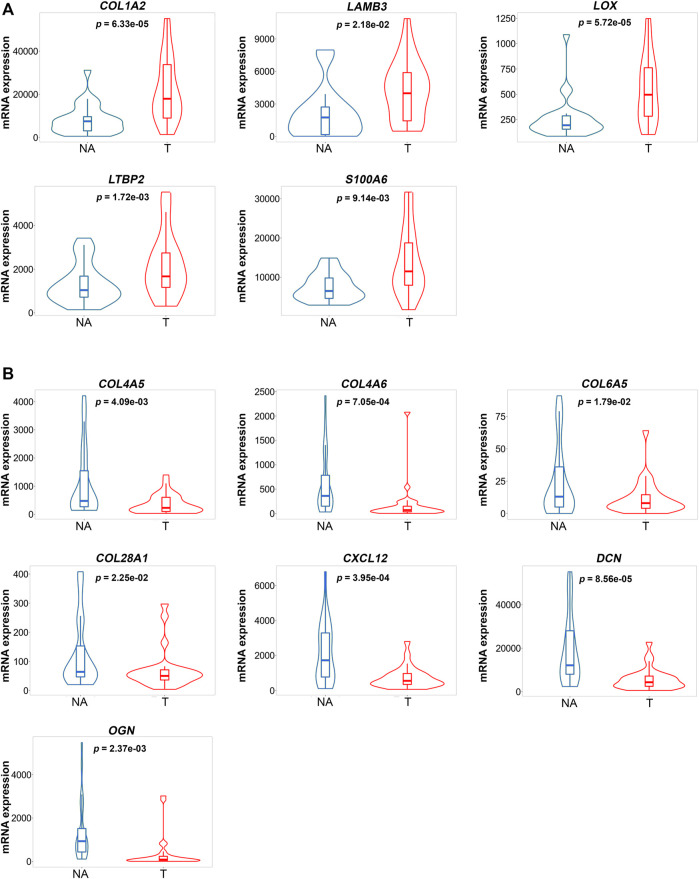
Gene expression violin plots of matrisome components differentially expressed in tumor ECM. Gastric cancer RNA-Seq expression from TCGA-STAD database comparing paired tumor (T) and normal adjacent (NA) tissue (*n* = 27). ECM genes significantly upregulated **(A)** or downregulated **(B)** in tumor samples. Plots were generated in the TNMplot platform [https://tnmplot.com/analysis/; (Bartha and Győrffy, 2021)]. Gene expression levels were normalized through DESeq2 (median-of-ratios method). Groups were compared using the Wilcoxon test and a *p* value below 0.05 was considered statistically significant.

We verified that out of the 24 differentially expressed components in tumor ECM (Figures 2B,C), 12 presented a transcriptomic profile in line with our proteomic results. In particular, we noticed that *COL1A2*, *LAMB3*, *LOX*, *LTBP2* and *S100A6* were significantly upregulated in tumor specimens, when compared with normal tissue (Figure 4A). The opposite pattern was detected for *COL4A5*, *COL4A6*, *COL6A5*, *COL28A1*, *CXCL12*, *DCN* and *OGN*, whose expression was found decreased in GC samples (Figure 4B).

**FIGURE 5:**
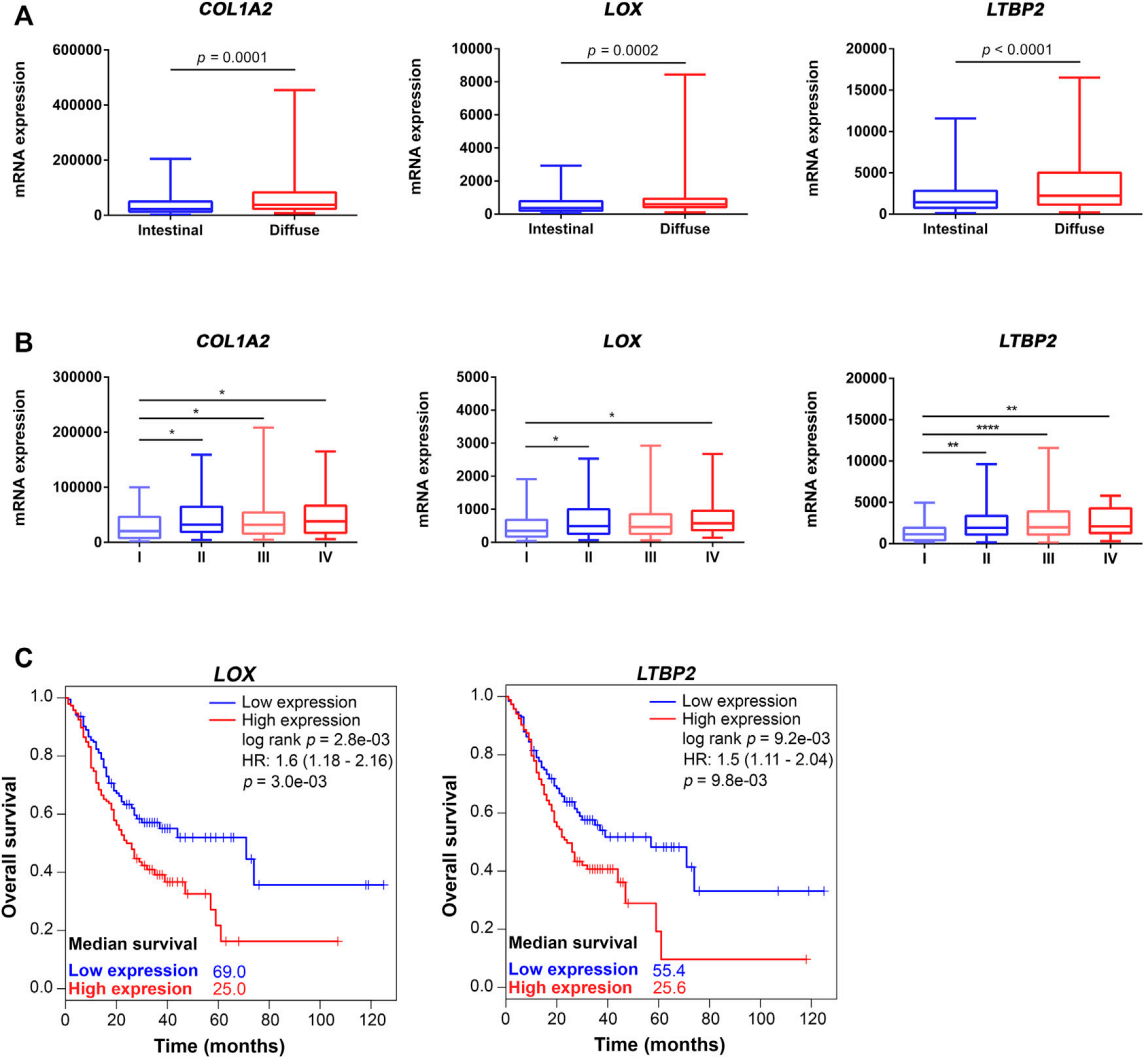
Association of differentially expressed ECM components with GC pathological features and overall survival. Increased expression of COL1A2, LOX and LTBP2 is significantly associated with diffuse type GC **(A)** and with high TNM stage **(B)**. Gene expression levels were normalized through DESeq2 (median-of-ratios method). Expression values were compared using student’s t test or one-way ANOVA followed by Tukey’s HSD test. *p < 0.05, **p < 0.01 and ****p < 0.0001. **(C)** Gastric cancer patients with increased tumor expression of LOX and LTBP2 have worse overall survival when compared with those exhibiting low expression. Survival between groups was compared using the Mantel-Cox test and log rank *p* value below 0.05 was considered statistically significant. Median survival refers to overall survival probability S(t) = 0.5. Vertical lines on Kaplan-Meier curves represent censored subjects.

We then evaluated the relationship of the validated candidates with available clinicopathological features (Supplementary Table S3). Remarkably, we observed that increased mRNA levels of *COL1A2*, *LOX* and *LTBP2* were significantly associated with diffuse-type gastric carcinomas and with higher TNM stage (Figures 5A,B).

To uncover the impact of candidate genes in patient overall survival, cases were divided into High and Low expression groups according to median expression of each of the 12 genes individually. Using Kaplan-Meier curves, we observed that increased expression of *LOX* and *LTBP2* was significantly associated with worst overall survival (log rank *p* < 0.05) (Figure 5C).

Consistent with what we observed for LOX and LTBP2, we found that COL10A1, which was the only tumor-specific protein, is strongly increased in tumor tissue (*p* = 5.93e-06), when compared with paired normal adjacent mucosa. Moreover, we observed an association between increased expression of COL10A1 and diffuse type gastric cancer, as well as with high TNM stage. Patients with tumors expressing high levels of COL10A1 also displayed lower overall survival, when compared with those harbouring tumors with low COL10A1 expression (Supplementary Figure S5).

These data highlight the role of core ECM components in gastric carcinogenesis and their clinical relevance as biomarkers of disease prognosis.

## Discussion

Gastric cancer remains a worldwide clinical burden, with scarce therapeutic options for patients who are often diagnosed at advanced stages of disease (Takahashi et al., 2013). The ECM is acknowledged to impact several cancer hallmarks and, in fact, matrisome changes have been described for a number of solid cancers (Naba et al., 2014a; Naba et al., 2014b; Acerbi et al., 2015; Laklai et al., 2016). To date, however, no proteomics-based matrisome signatures have been described for GC.

Research on the roles of ECM in cancer has traditionally focused on single or multiple pre-established combinations of ECM components. In the last decade, advances in decellularization strategies have attempted to overcome this limitation and obtain reconstituted ECM *in vitro* (Hoshiba, 2019). Herein, we present a comprehensive analysis of the gastric matrisome and the identification of key constituents using tissue-derived decellularized ECM, which could represent useful biomarkers for GC management. To the best of our knowledge, this is the first study that characterizes molecular alterations of gastric tumor ECM, considering both adjacent tissue and distant mucosa. Indeed, most studies addressing tumor/matched normal pairs use normal tissue surrounding the lesion. Although histologically normal, evidence indicate that these adjacent tissues display alterations in gene expression related to fibrosis, wound healing, epithelial-to-mesenchymal transition, and ECM remodeling (Troester et al., 2009; Trujillo et al., 2011; Casbas-Hernandez et al., 2015). Therefore, to obtain a thorough characterization of gastric matrisome alterations that may impact tumor development, we have used paired distant mucosa as our main control.

Through a decellularization approach that combined the use of a hypotonic buffer, an anionic surfactant, and DNase, we have obtained ECM-enriched samples of gastric adenocarcinomas as well as paired normal adjacent and normal distant mucosa, which were then processed for high resolution mass spectrometry. For the definition of each tissue matrisome, we have considered only proteins present in at least 1/3 of patient samples. This resulted in the identification of a total of 157 ECM proteins, 93 of which were core matrisome proteins and 64 ECM-associated proteins. To define the gastric matrisome, we identified a common ECM signature among the three tissues composed of 142 proteins. These, along with 11 more proteins that were detected in the normal distant mucosa (alone, or in combination with one of the other tissues) constitute the gastric matrisome.

Interestingly, our proteomic analysis has uncovered three proteins that were present either in tumor ECM alone (COL10A1) or in tumor and adjacent mucosa (EFEMP2 and vWF) and were absent in most distant mucosa samples. Corroborating our results, Necula and colleagues reported increased levels of COL10A1 in the plasma of GC patients, which were associated with poor overall survival (Necula et al., 2020). With respect to EFEMP2, our results are consistent with previous reports of EFEMP2 mRNA overexpression associated with pathohistological alterations and overall survival in GC (Chen et al., 2018). Regarding vWF expression in GC, it was found upregulated both at the mRNA and protein levels, and increased vWF antigen levels correlated with disease severity (Yang et al., 2015). We propose that these three proteins hold great potential as clinical biomarkers for early detection of GC and could be more useful than currently used markers, such as CEA and pepsinogen, which lack sensitivity and specificity (Battaglin et al., 2018; Necula et al., 2019).

Given that most ECM proteins were ubiquitously expressed in normal and tumor tissues, we postulated that the major molecular alterations occurring in gastric mucosa during transformation could be related to differential expression of its components. To disclose these modifications, we have compared the matrisome of gastric adenocarcinoma to those of normal distant and adjacent mucosa, identifying 24 differentially expressed ECM proteins, of which eight were upregulated and 16 were downregulated in tumor tissues. Overexpressed in tumor matrix were proteins involved in ECM organization such as LOX, LAMB3, ADAMTSL1 and FBN2. Indeed, LOX has been reported to play a major role in ECM remodeling by establishing covalent crosslinks between collagen and elastic fibers that lead to ECM fibrosis and set the grounds for adherence and colonization of cancer cells (Zhao et al., 2019). LAMB3 has been shown to activate the PI3K/Akt signaling pathway, promoting cancer cell proliferation, migration and invasion *in vitro* (Zhang et al., 2019). ADAMTS-like proteins are a subgroup of the ADAMTS family that lack the catalytic domain (Porter et al., 2005) and have been described to play a role in microfibril formation through FBN1 (fibrillin-1) and FBN2 binding (Tsutsui et al., 2010). Fibrillin microfibrils not only award elastic properties to the matrix, but also bind LTBP proteins, controlling bioavailability of TGF-β superfamily molecules and, thus, regulating growth factor signaling (Chaudhry et al., 2007). Although there is little information concerning ADAMTSL1, we propose that alterations in both this component and FBN2 will impact cell-matrix interactions and influence cellular fate towards an oncogenic phenotype. Likewise, the increase of LTBPs is expected to enhance GC cells metastatic ability by promoting TGF-β-induced EMT (Chandramouli et al., 2011).

Among the downregulated proteins, we identified important basement membrane components, namely COL4A5, COL4A6, COL28A1, NID1, and LAMA5. The basement membrane is a specialized form of ECM that supports the epithelial layer and acts as a barrier that cancer cells need to breach to invade the surrounding stroma (Glentis et al., 2014). Baba and colleagues (Baba et al., 2007) have reported the gradual disappearance of type IV collagen alpha chains from the basement membrane which, along with changes in other structural components like laminins and nidogen, weaken this complex, favoring cancer cell invasion and progression. In addition, the proteoglycan decorin was found to be downregulated in the identified tumor matrisome, consistent with its role as a negative regulator of TGF-β signaling (Troup et al., 2003). In fact, our data suggest that during the gastric carcinogenic process, there is an increase in the levels of ECM components responsible for growth factor binding, particularly to TGF-β superfamily molecules, potentiating TGF-β-driven tumor growth and invasion, evasion of immune surveillance, cancer cell dissemination and future metastasis (Massagué, 2008). In contrast, downregulated proteins are mainly involved in cell adhesive properties, metabolism of glycosaminoglycans, and negative regulation of endopeptidase activity, in frame with the modulation of a pro-tumorigenic microenvironment (Wei et al., 2020; Zhang and Lin, 2021).

To corroborate our findings, we have analysed mRNA expression of identified candidates in an independent GC cohort available at the TGCA. Importantly, the expression profile of tumor and normal tissues was confirmed for 12 genes. Further, analysis of the association between those ECM components and GC clinicopathological features revealed that increased expression of *COL1A2*, *LOX* and *LTBP2* significantly correlated with high tumor stage. Elevated *COL1A2* levels were previously reported in patients with advanced disease stages (Yasui et al., 2004). Interestingly, co-expression of *COL1A2* and *LOX*—whose expression in the TCGA-STAD database is highly correlated (Pearson’s correlation coefficient = 0.87, *p*-value = 6.21e-83)—promotes cancer drug-resistance by increasing collagen cross linking, consequently, stiffening the extracellular matrix and blocking drug diffusion (Di Paolo and Bocci, 2007; Sterzyńska et al., 2018). LTBP2 is known to regulate EMT markers and improve migratory and invasive capacities of GC cells through a mechanism independent of TGF-β signaling (Saharinen and Keski-Oja, 2000). Accordingly, we verified that high levels of *LOX* and *LTBP2* were also associated with poorer patient outcome, when compared with those exhibiting low expression.

In conclusion, we have identified a set of ECM constituents that, to our knowledge, characterize for the first time the matrisome of gastric normal and tumor contexts. Of note, we found that quantitative changes of LOX, LTBP2 and COL1A2 contribute to disease progression and patient poor prognosis. These data provide evidence that ECM components should be explored as novel diagnostic and prognostic biomarkers in GC. Moreover, an essential topic within this research area will be to further advance the functional significance of the relevant ECM components. The understanding of ECM dynamics and the identification of its regulators will be key for the development of innovative therapeutic approaches.

## Data Availability

The mass spectrometry proteomics data have been deposited to the ProteomeXchange Consortium via the PRIDE (Perez-Riverol et al., 2019) partner repository (https://www.ebi.ac.uk/pride/archive/) with the dataset identifier PXD029782 and 10.6019/PXD029782.
