# Supplementary material for: Proteomic Identification of a Gastric Tumor ECM Signature Associated With Cancer Progression
Source: Front Mol Biosci. 2022 Mar 1;9:818552. doi: 10.3389/fmolb.2022.818552 (PMC8942767; doi:10.3389/fmolb.2022.818552)
Supplement: Supplementary file 1 [file Table1.PDF]

Supplementary Table 1. Summary of clinicopathological features from nine gastric cancer cases submitted to proteomic analysis.

**Total no. of patients**	**9**
**Age, years**	
Median (range)	62 (39-84)
**Sex**	
Male	7
Female	2
Tumor site	Antrum
**WHO classification**	
Tubular	5
Mixed	4
**Laurén classification**	
Intestinal	4
Mixed	4
Indeterminate	1
**Growth pattern**	
Expansive	2
Infiltrative	5
Indeterminate	2
**TNM stage**	
I/II	4
III/IV	5
**T category**	
T1/T2	2
T3/T4	7
**N category**	
N0	4
N+	5
**Resection margins**	
R0	9
R1/R2	0
**Vascular invasion**	
Absent	5
Present	4

WHO, World Health Organization; *TNM*, Tumor Node Metastasis
