# Supplementary figures and images for: Proteomic Identification of a Gastric Tumor ECM Signature Associated With Cancer Progression
Source: Front Mol Biosci. 2022 Mar 1;9:818552. doi: 10.3389/fmolb.2022.818552 (PMC8942767; doi:10.3389/fmolb.2022.818552)

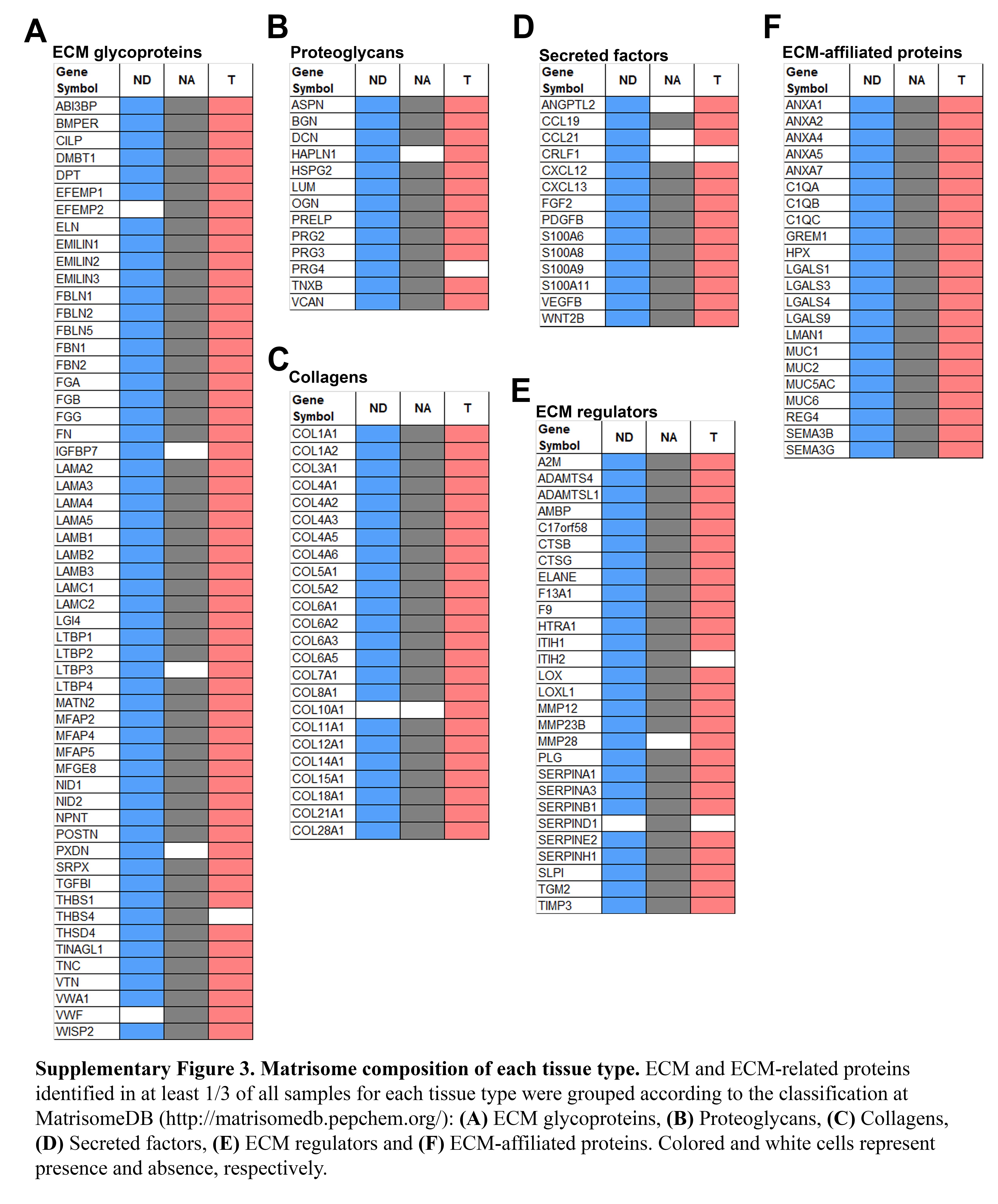

Supplement: Supplementary file 2 [file Image3.tif]

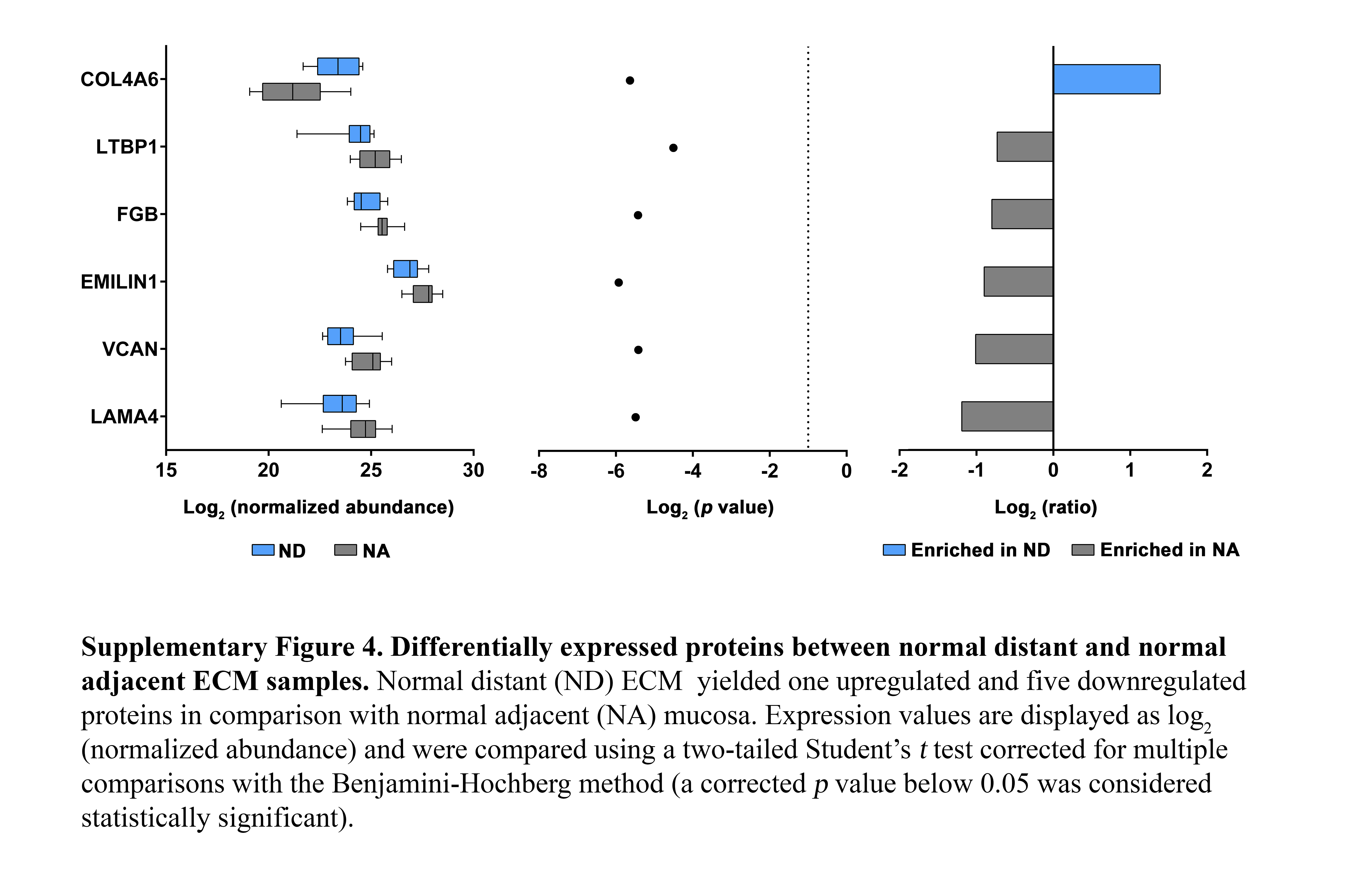

Supplement: Supplementary file 3 [file Image4.TIF]

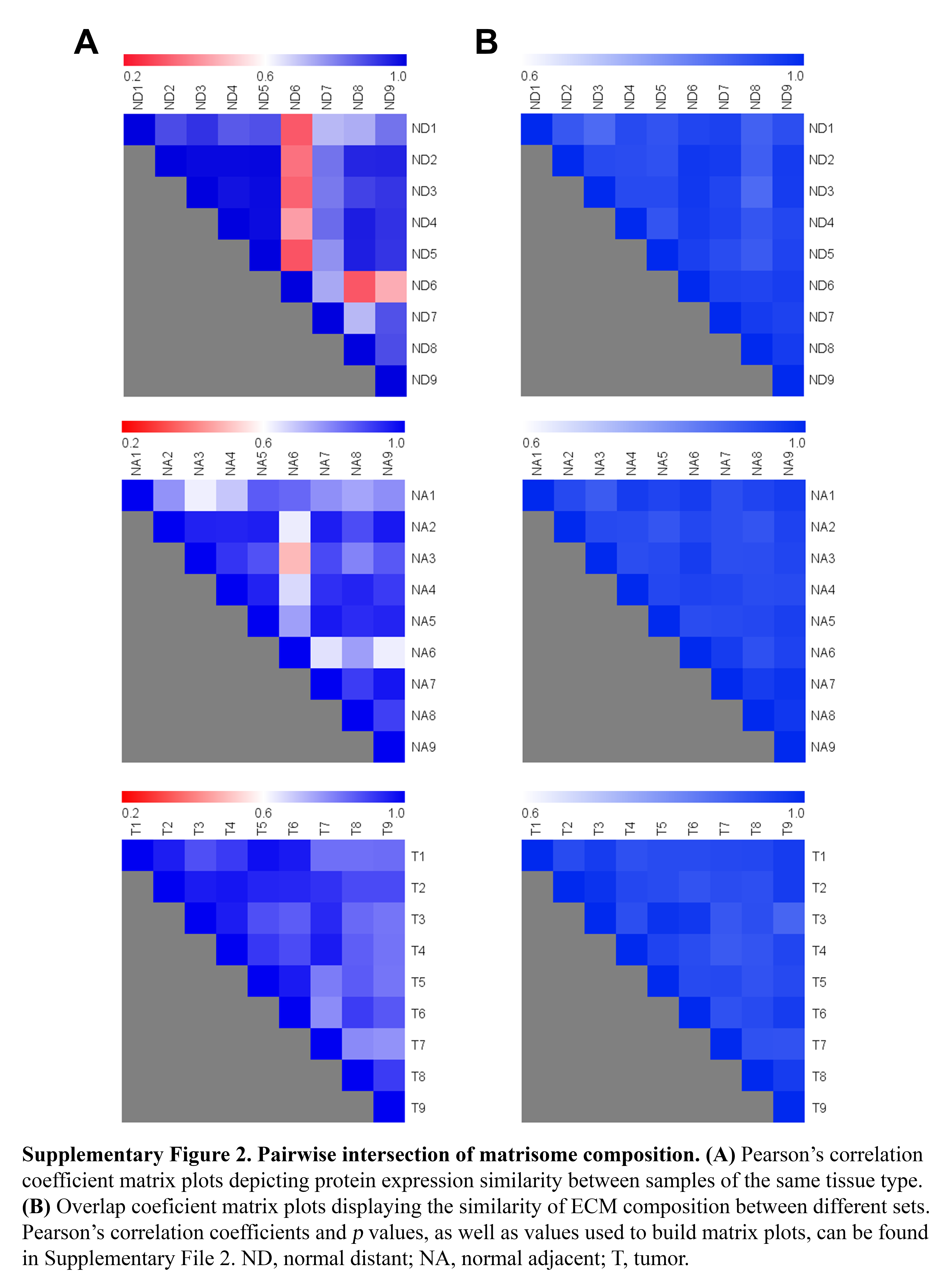

Supplement: Supplementary file 5 [file Image2.tif]

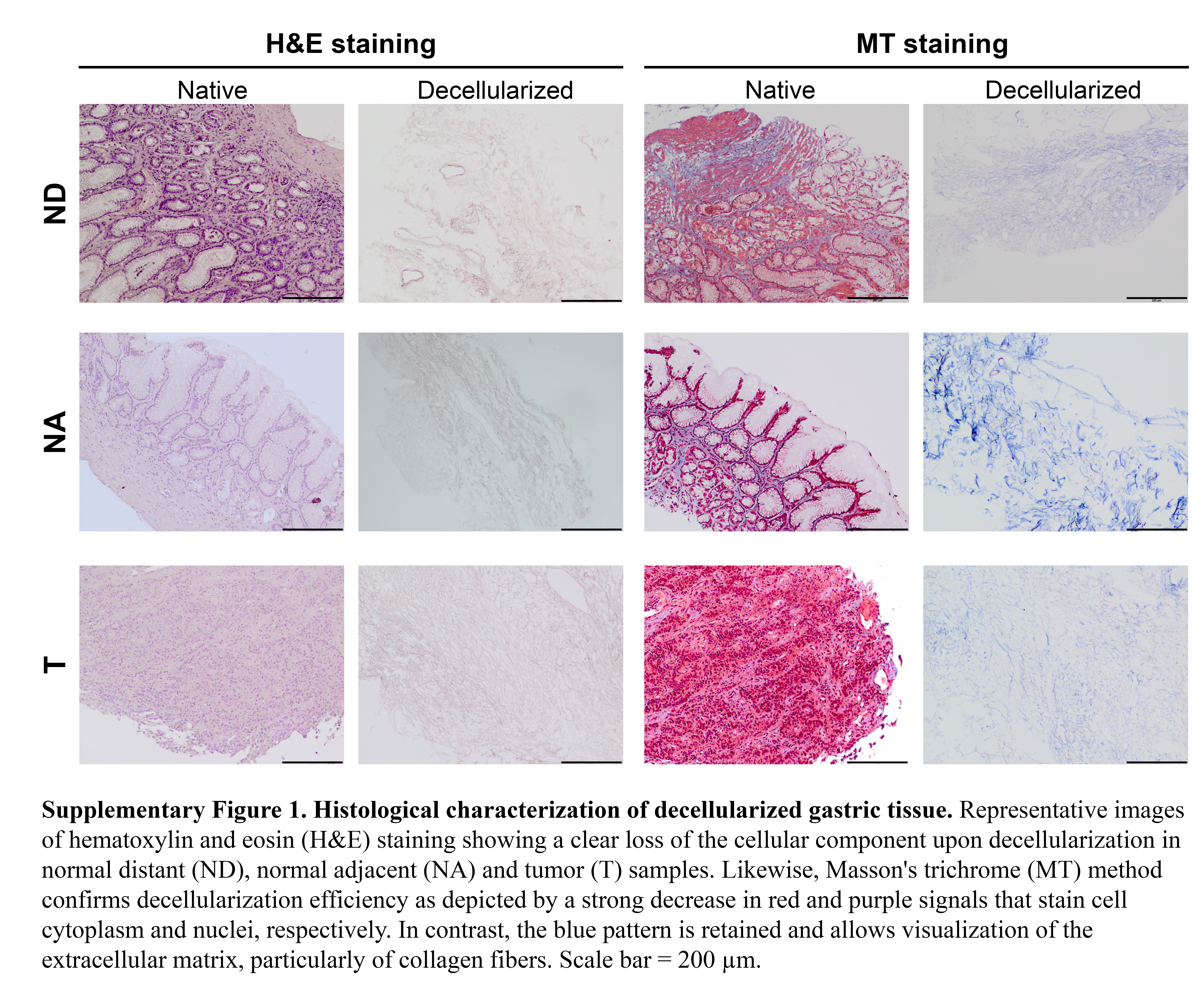

Supplement: Supplementary file 6 [file Image1.TIF]

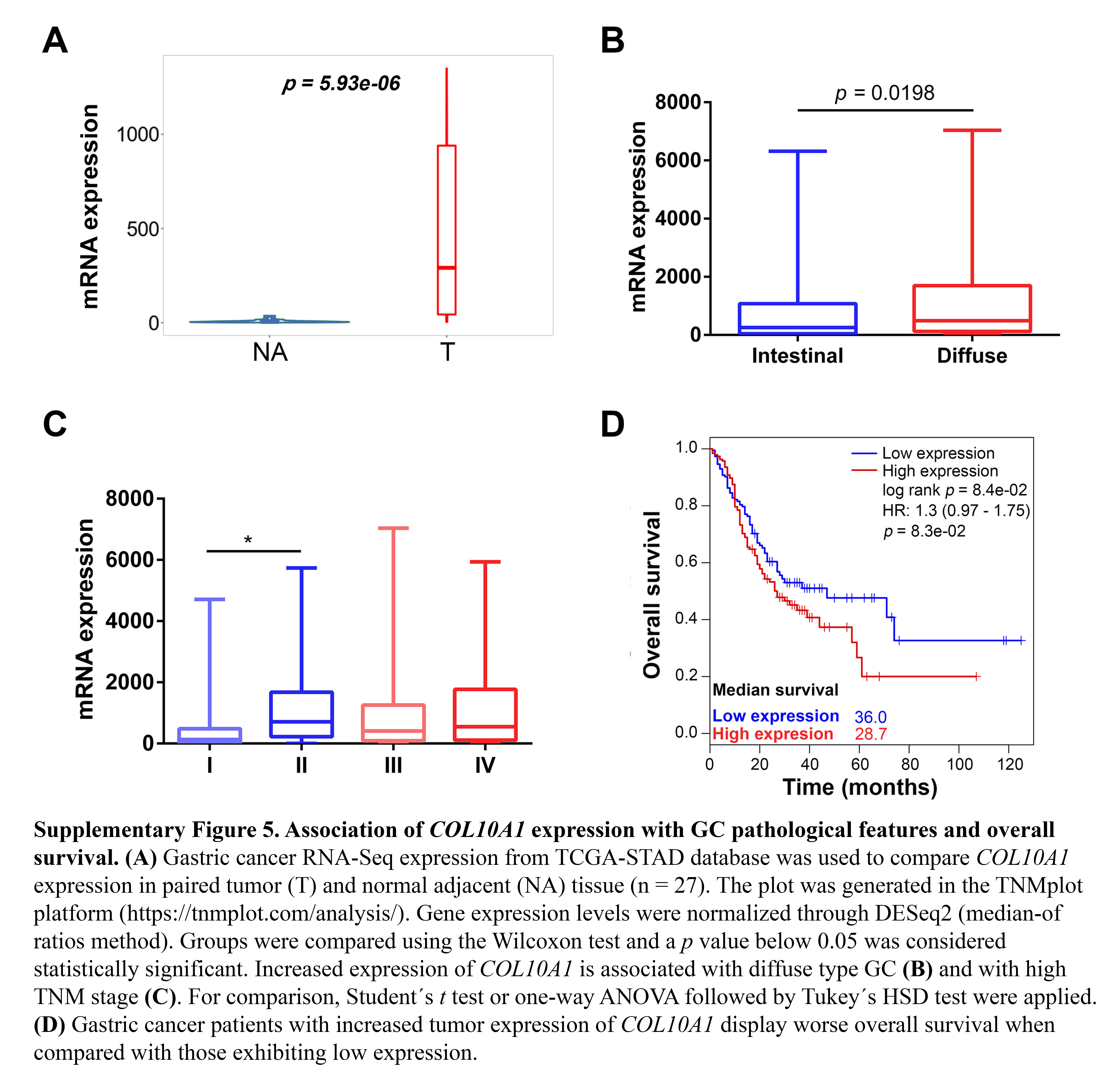

Supplement: Supplementary file 8 [file Image5.tif]
