# Supplementary material for: Proteomic Identification of a Gastric Tumor ECM Signature Associated With Cancer Progression
Source: Front Mol Biosci. 2022 Mar 1;9:818552. doi: 10.3389/fmolb.2022.818552 (PMC8942767; doi:10.3389/fmolb.2022.818552)
Supplement: Supplementary file 4 [file Table2.PDF]

Supplementary Table 2. Functional and pathway enrichment analysis of the 24 differentially expressed proteins in decellularized tumor ECM.

**Biological Process**						
**Term ID**	**Term description**	**Observed gene count**	**Background gene count**	**Enrichment strength**	**False discovery rate**	**Matching proteins in our network (labels)**
GO:0035583	sequestering of TGFbeta in extracellular matrix	2	3	2.74	1.10e-03	LTBP1,FBN2
GO:0031581	hemidesmosome assembly	2	12	2.13	4.40e-03	LAMC1,LAMB3
GO:0042340	keratan sulfate catabolic process	2	12	2.13	4.40e-03	OGN,LUM
GO:0030199	collagen fibril organization	3	39	1.8	1.30e-03	LOX,LUM,COL1A2
GO:0007044	cell-substrate junction assembly	3	40	1.79	1.30e-03	LAMA5,LAMC1,LAMB3
GO:0018146	keratan sulfate biosynthetic process	2	29	1.75	1.52e-02	OGN,LUM
GO:0006027	glycosaminoglycan catabolic process	4	62	1.72	1.90e-04	DCN,OGN,LUM,HSPG2
GO:0044273	sulfur compound catabolic process	3	53	1.66	2.20e-03	DCN,OGN,LUM
GO:0034446	substrate adhesion-dependent cell spreading	2	38	1.63	2.17e-02	LAMA5,LAMC1
GO:0030198	extracellular matrix organization	13	296	1.55	5.68e-15	DCN,LOX,LAMA5,LAMC1,NID1,LUM,COL6A3,COL1A2,COL4A5,HSPG2,LAMB3,COL4A6,FBN2
GO:0035904	aorta development	2	49	1.52	2.99e-02	LOX,LTBP1
GO:0006024	glycosaminoglycan biosynthetic process	4	101	1.51	7.90e-04	DCN,OGN,LUM,HSPG2
GO:1903510	mucopolysaccharide metabolic process	3	109	1.35	8.80e-03	DCN,OGN,LUM
GO:0090288	negative regulation of cellular response to growth factor stimulus	3	137	1.25	1.52e-02	DCN,LTBP1,FBN2
GO:0031589	cell-substrate adhesion	3	162	1.18	2.12e-02	LAMA5,LAMC1,NID1
GO:0044272	sulfur compound biosynthetic process	3	172	1.15	2.35e-02	DCN,OGN,LUM

GO:0010951	negative regulation of endopeptidase activity	4	242	1.13	6.70e-03	AMBP,COL6A3,SLPI,COL28A1
GO:0007178	transmembrane receptor protein serine/threonine kinase signaling pathway	3	189	1.11	2.90e-02	LTBP2,COL1A2,LTBP1
GO:0007155	cell adhesion	10	843	0.99	7.97e-06	LAMA5,LAMC1,NID1,AMBP,COL6A5,COL6A3,LAMB3,COL4A6,CXCL12,COL28A1
GO:0001568	blood vessel development	5	464	0.94	6.80e-03	LOX,LAMA5,COL1A2,HSPG2,LTBP1
GO:0097435	supramolecular fiber organization	4	383	0.93	2.27e-02	LOX,LTBP2,LUM,COL1A2
GO:0000904	cell morphogenesis involved in differentiation	5	498	0.91	8.40e-03	LAMA5,LAMC1,OGN,S100A6,CXCL12
GO:0001501	skeletal system development	4	457	0.85	3.55e-02	LUM,COL1A2,WNT2B,FBN2
GO:0032989	cellular component morphogenesis	5	720	0.75	2.90e-02	LAMA5,LAMC1,OGN,S100A6,CXCL12
GO:0043086	negative regulation of catalytic activity	5	809	0.7	4.20e-02	DCN,AMBP,COL6A3,SLPI,COL28A1
GO:0009653	anatomical structure morphogenesis	12	1992	0.69	1.90e-04	DCN,LOX,LAMA5,LAMC1,OGN,COL1A2,S100A6,WNT2B,HSPG2,LAMB3,CXCL12,FBN2
GO:0051248	negative regulation of protein metabolic process	6	1075	0.66	2.90e-02	DCN,AMBP,COL6A3,SLPI,HSPG2,COL28A1
GO:0048513	animal organ development	12	2926	0.52	2.50e-03	DCN,LOX,LAMA5,NID1,LUM,COL6A3,COL1A2,WNT2B,HSPG2,CXCL12,LTBP1,FBN2
GO:0048731	system development	14	4144	0.44	2.90e-03	DCN,LOX,LAMA5,OGN,NID1,LUM,COL6A3,COL1A2,

						S100A6,WNT2B,HSPG2,CXCL12,LTBP1,FBN2
GO:0016043	cellular component organization	17	5163	0.43	7.90e-04	DCN,LOX,LAMA5,LAMC1,LTBP2,OGN,NID1,LUM,COL6A3,COL1A2,COL4A5,S100A6,HSPG2,LAMB3,COL4A6,CXCL12,FBN2
GO:0048856	anatomical structure development	16	5085	0.41	2.10e-03	DCN,LOX,LAMA5,LAMC1,OGN,NID1,LUM,COL6A3,COL1A2,S100A6,WNT2B,HSPG2,LAMB3,CXCL12,LTBP1,FBN2
GO:0007275	multicellular organism development	15	4726	0.41	2.80e-03	DCN,LOX,LAMA5,OGN,NID1,LUM,COL6A3,COL1A2,S100A6,WNT2B,HSPG2,LAMB3,CXCL12,LTBP1,FBN2
GO:0032501	multicellular organismal process	16	6507	0.30	1.85e-02	DCN,LOX,LAMA5,OGN,NID1,AMBP,LUM,COL6A3,COL1A2,S100A6,WNT2B,HSPG2,LAMB3,CXCL12,LTBP1,FBN2
**Molecular Function**						
**Term ID**	**Term description**	**Observed gene count**	**Background gene count**	**Enrichment strength**	**False discovery rate**	**Matching proteins in your network (labels)**
GO:0005201	extracellular matrix structural constituent	7	73	1.89	5.31e-10	LAMC1,COL6A5,LUM,COL1A2,COL4A5,COL4A6,FBN2
GO:0005518	collagen binding	3	61	1.60	1.20e-03	DCN,NID1,LUM
GO:0004867	serine-type endopeptidase inhibitor activity	4	94	1.54	2.20e-04	AMBP,COL6A3,SLPI,COL28A1

GO:0050840	extracellular matrix binding	2	51	1.50	1.51e-02	DCN,NID1
GO:0005178	integrin binding	3	122	1.30	4.30e-03	LAMA5,HSPG2,CXCL12
GO:0019838	growth factor binding	3	126	1.29	4.40e-03	LTBP2,COL1A2,LTBP1
GO:0004857	enzyme inhibitor activity	5	388	1.02	1.20e-03	DCN,AMBP,COL6A3,SLPI, COL28A1
GO:0005198	structural molecule activity	8	679	0.98	5.50e-05	LAMC1,COL6A5,LUM,COL1A2,COL4A5,LAMB3,COL4A6,FBN2
GO:0005509	calcium ion binding	6	700	0.84	1.70e-03	LTBP2,NID1,S100A6,HSPG2,LTBP1,FBN2
GO:0044877	protein-containing complex binding	8	968	0.83	3.70e-04	DCN,LAMA5,NID1,AMBP, LUM,HSPG2,LAMB3,CXCL12
GO:0098772	molecular function regulator	7	1793	0.50	3.26e-02	DCN,OGN,AMBP,COL6A3, SLPI,CXCL12,COL28A1
**Cellular Component**						
**Term ID**	**Term description**	**Observed gene count**	**Background gene count**	**Enrichment strength**	**False discovery rate**	**Matching proteins in your network (labels)**
GO:0031012	extracellular matrix	15	283	1.64	3.64e-20	LOX,LAMA5,LAMC1,LTBP2,NID1,LUM,COL6A3,COL1A2,COL4A5,HSPG2,LAMB3,COL4A6,COL28A1,LTBP1,FBN2
GO:0005576	extracellular region	21	2505	0.83	1.10e-14	LOX,LAMA5,LAMC1,LTBP2,NID1,AMBP,COL6A5,LUM,COL6A3,COL1A2,COL4A5,SLPI,WNT2B,HSPG2, ADAMTSL1,LAMB3,COL4A6,CXCL12,COL28A1,LTBP1,FBN2

GO:0005581	collagen trimer	9	88	1.92	3.31e-14	DCN,LOX,COL6A5,LUM,COL6A3,COL1A2,COL4A5,COL4A6,COL28A1
GO:0062023	collagen-containing extracellular matrix	10	144	1.75	3.31e-14	LAMA5,LAMC1,NID1,LUM,COL1A2,COL4A5,HSPG2,LAMB3,COL4A6,COL28A1
GO:0005604	basement membrane	8	91	1.86	3.64e-12	LAMA5,LAMC1,NID1,COL4A5,HSPG2,LAMB3,COL4A6,COL28A1
GO:0005788	endoplasmic reticulum lumen	8	299	1.34	2.72e-08	LAMC1,COL6A3,COL1A2,COL4A5,ADAMTSL1,COL4A6,COL28A1,LTBP1
GO:0098644	complex of collagen trimers	4	19	2.23	1.79e-07	LUM,COL1A2,COL4A5,COL4A6
GO:0043256	laminin complex	3	8	2.49	2.75e-06	LAMA5,LAMC1,LAMB3
GO:0043202	lysosomal lumen	4	94	1.54	5.34e-05	DCN,OGN,LUM,HSPG2
GO:0005796	Golgi lumen	4	101	1.51	5.51e-05	DCN,OGN,LUM,HSPG2
GO:0099081	supramolecular polymer	8	880	0.87	5.51e-05	DCN,LUM,COL6A3,COL1A2,COL4A5,COL4A6,LTBP1,FBN2
GO:0005589	collagen type VI trimer	2	3	2.74	9.16e-05	DCN,COL6A3
GO:0005610	laminin-5 complex	2	3	2.74	9.16e-05	LAMA5,LAMB3
GO:0043259	laminin-10 complex	2	3	2.74	9.16e-05	LAMA5,LAMC1
GO:0043260	laminin-11 complex	2	3	2.74	9.16e-05	LAMA5,LAMC1
GO:0005587	collagen type IV trimer	2	6	2.43	1.80e-04	COL4A5,COL4A6
GO:0001527	microfibril	2	10	2.21	3.40e-04	LTBP1,FBN2
GO:0005583	fibrillar collagen trimer	2	11	2.17	3.80e-04	LUM,COL1A2
GO:0012505	endomembrane system	14	4347	0.42	4.50e-04	DCN,LAMC1,OGN,LUM,COL6A3,COL1A2,COL4A5,SLPI,S100A6,HSPG2,ADAM

						TSL1,COL4A6,COL28A1,LTBP1
GO:0032991	protein-containing complex	14	4792	0.38	1.30e-03	DCN,LOX,LAMA5,LAMC1,COL6A5,LUM,COL6A3,COL1A2,COL4A5,HSPG2,LAMB3,COL4A6,COL28A1,LTBP1
GO:0099512	supramolecular fiber	6	873	0.75	1.60e-03	DCN,LUM,COL6A3,COL1A2,LTBP1,FBN2
GO:0005615	extracellular space	6	1134	0.63	5.80e-03	LOX,LAMA5,LAMC1,COL1A2,SLPI,WNT2B
GO:0070013	intracellular organelle lumen	13	5162	0.31	9.50e-03	DCN,LAMC1,OGN,LUM,COL6A3,COL1A2,COL4A5,SLPI,HSPG2,ADAMTSL1,COL4A6,COL28A1,LTBP1
**Protein Domains and Features**						
**Term ID**	**Term description**	**Observed gene count**	**Background gene count**	**Enrichment strength**	**False discovery rate**	**Matching proteins in your network (labels)**
IPR000034	Laminin IV	3	8	2.49	4.29e-06	LAMA5,LAMC1,HSPG2
IPR017878	TB domain	3	9	2.43	4.76e-06	LTBP2,LTBP1,FBN2
IPR001442	Collagen IV, non-collagenous	2	6	2.43	1.80e-04	COL4A5,COL4A6
IPR036954	Collagen IV, non-collagenous domain superfamily	2	6	2.43	1.80e-04	COL4A5,COL4A6
IPR036773	TGF-beta binding (TB) domain superfamily	3	10	2.39	4.76e-06	LTBP2,LTBP1,FBN2
IPR008211	Laminin, N-terminal	3	16	2.18	1.39e-05	LAMA5,LAMC1,LAMB3
IPR038684	Laminin, N-terminal domain superfamily	3	16	2.18	1.39e-05	LAMA5,LAMC1,LAMB3
IPR020901	Proteinase inhibitor I2, Kunitz, conserved site	3	18	2.13	1.56e-05	AMBP,COL6A3,COL28A1

IPR002223	Pancreatic trypsin inhibitor Kunitz domain	3	19	2.11	1.65e-05	AMBP,COL6A3,COL28A1
IPR036880	Pancreatic trypsin inhibitor Kunitz domain superfamily	3	19	2.11	1.65e-05	AMBP,COL6A3,COL28A1
IPR002049	Laminin EGF domain	4	36	1.96	3.18e-06	LAMA5,LAMC1,HSPG2,LAMB3
IPR026823	Complement Clr-like EGF domain	3	27	1.96	3.63e-05	NID1,LTBP1,FBN2
IPR008160	Collagen triple helix repeat	6	76	1.81	4.58e-08	COL6A5,COL6A3,COL1A2,COL4A5,COL4A6,COL28A1
IPR001881	EGF-like calcium-binding domain	5	121	1.53	4.76e-06	LTBP2,NID1,HSPG2,LTBP1,FBN2
IPR018097	EGF-like calcium-binding, conserved site	4	96	1.53	3.63e-05	LTBP2,NID1,LTBP1,FBN2
IPR000152	EGF-type aspartate/asparagine hydroxylation site	4	100	1.51	3.72e-05	LTBP2,NID1,LTBP1,FBN2
IPR002035	von Willebrand factor, type A	3	76	1.51	4.90e-04	COL6A5,COL6A3,COL28A1
IPR000372	Leucine-rich repeat N-terminal domain	2	54	1.48	7.50e-03	DCN,LUM
IPR001791	Laminin G domain	2	58	1.45	8.20e-03	LAMA5,HSPG2
IPR009030	Growth factor receptor cysteine-rich domain superfamily	4	125	1.42	8.20e-05	LTBP2,NID1,LTBP1,FBN2
IPR000742	EGF-like domain	7	225	1.40	3.52e-07	LAMA5,LAMC1,LTBP2,NID1,HSPG2,LTBP1,FBN2
IPR013032	EGF-like, conserved site	6	193	1.40	3.18e-06	LTBP2,NID1,HSPG2,LAMB3,LTBP1,FBN2
IPR036465	von Willebrand factor A-like domain superfamily	3	97	1.40	9.30e-04	COL6A5,COL6A3,COL28A1
IPR016187	C-type lectin fold	2	108	1.18	2.39e-02	COL4A5,COL4A6
IPR003591	Leucine-rich repeat, typical subtype	3	178	1.14	5.00e-03	DCN,OGN,LUM
IPR013098	Immunoglobulin I-set	2	132	1.09	3.36e-02	HSPG2,ADAMTSL1

IPR001611	Leucine-rich repeat	3	259	0.98	1.26e-02	DCN,OGN,LUM
IPR032675	Leucine-rich repeat domain superfamily	3	312	0.89	2.02e-02	DCN,OGN,LUM
**KEGG pathway**						
**Term ID**	**Term description**	**Observed gene count**	**Background gene count**	**Enrichment strength**	**False discovery rate**	**Matching proteins in your network (labels)**
hsa04512	ECM-receptor interaction	9	81	1.96	2.61e-14	LAMA5,LAMC1,COL6A5, COL6A3,COL1A2,COL4A5 ,HSPG2,LAMB3,COL4A6
hsa04510	Focal adhesion	8	197	1.52	1.40e-09	LAMA5,LAMC1,COL6A5, COL6A3,COL1A2,COL4A5 ,LAMB3,COL4A6
hsa05165	Human papillomavirus infection	9	317	1.36	1.40e-09	LAMA5,LAMC1,COL6A5, COL6A3,COL1A2,COL4A5 ,WNT2B,LAMB3,COL4A6
hsa05146	Amoebiasis	6	94	1.72	1.66e-08	LAMA5,LAMC1,COL1A2, COL4A5,LAMB3,COL4A6
hsa04151	PI3K-Akt signaling pathway	8	348	1.27	4.39e-08	LAMA5,LAMC1,COL6A5, COL6A3,COL1A2,COL4A5 ,LAMB3,COL4A6
hsa04974	Protein digestion and absorption	5	90	1.66	5.56e-07	COL6A5,COL6A3,COL1A2 ,COL4A5,COL4A6
hsa05222	Small cell lung cancer	5	92	1.65	5.56e-07	LAMA5,LAMC1,COL4A5, LAMB3,COL4A6
hsa05200	Pathways in cancer	7	515	1.04	9.36e-06	LAMA5,LAMC1,COL4A5, WNT2B,LAMB3,COL4A6, CXCL12
hsa05205	Proteoglycans in cancer	4	195	1.22	3.60e-04	DCN,LUM,WNT2B,HSPG2
hsa04933	AGE-RAGE signaling pathway in diabetic complications	3	98	1.40	8.70e-04	COL1A2,COL4A5,COL4A6

hsa05145	Toxoplasmosis	3	109	1.35	1.10e-03	LAMA5,LAMC1,LAMB3
hsa04926	Relaxin signaling pathway	3	130	1.27	1.60e-03	COL1A2,COL4A5,COL4A6
hsa04350	TGF-beta signaling pathway	2	83	1.29	1.30e-02	DCN,LTBP1
