# Supplementary material for: Proteomic Identification of a Gastric Tumor ECM Signature Associated With Cancer Progression
Source: Front Mol Biosci. 2022 Mar 1;9:818552. doi: 10.3389/fmolb.2022.818552 (PMC8942767; doi:10.3389/fmolb.2022.818552)
Supplement: Supplementary file 7 [file Table3.PDF]

Supplementary Table 3. Summary of clinicopathological features of 383 gastric cancer cases retrieved from The Cancer Genome Atlas (Cancer Genome Atlas Research Network, 2014; Liu et al., 2018).

	**STAD-TCGA**
**Total no. of patients**	383
**Age, years**	
Median (range)	67.7 (30 - 90)
< median	190
≥ median	190
NA	3
**Sex**	
Male	252
Female	131
**Laurén classification**	
Diffuse	77
Intestinal	161
NOS	145
**TNM stage**	
I	51
II	115
III	162
IV	39
NA	16
**T stage**	
T1	20
T2	81
T3	168
T4	106
TX	8
**N stage**	
N0	111
N1	106
N2	74
N3	74
NX	17
NA	1
**M stage**	
M0	339
M1	25
MX	19

STAD, Stomach adenocarcinoma; TCGA, The Cancer Genome Atlas; *TNM*, Tumor Node Metastasis.

References

Cancer Genome Atlas Research Network (2014). Comprehensive molecular characterization of gastric adenocarcinoma. *Nature* 513(7517)**,** 202-209. doi: 10.1038/nature13480.

Liu, Y., Sethi, N.S., Hinoue, T., Schneider, B.G., Cherniack, A.D., Sanchez-Vega, F., et al. (2018). Comparative Molecular Analysis of Gastrointestinal Adenocarcinomas. *Cancer Cell* 33(4)**,** 721-735.e728. doi: 10.1016/j.ccell.2018.03.010.
